# Clinical Characteristics and Reproduction Number of Coronavirus Disease (COVID-19) Cases in Markazi Province in Iran

**DOI:** 10.30476/IJCBNM.2020.86339.1338

**Published:** 2021-01

**Authors:** Rahmatollah Moradzadeh, Maryam Zamanian, Saeed Amini, Masoumeh Kalantari, Javad Nazari

**Affiliations:** 1 Department of Epidemiology, School of Health, Arak University of Medical Sciences, Arak, Iran; 2 Department of Health Services management, School of Health, Arak University of Medical Sciences, Arak, Iran; 3 Medical Science Researcher, Arak University of Medical sciences, Arak, Iran; 4 Department of Pediatrics, School of Medicine, Arak University of Medical Sciences, Arak, Iran

**Keywords:** COVID-19, Demographic and clinical characteristics, Epidemiology, Iran, Reproduction number

## Abstract

**Background:**

The first case of Coronavirus Disease (COVID-19) was reported in Iran on February 19, 2020. This study aimed to assess the characteristics and reproduction number (R) of COVID-19 in Markazi province in Iran.

**Methods:**

This is a cross-sectional study. Confirmed cases (N=2430) in the regions covered by Arak University of Medical Sciences from Feb 20 to Aug 26, 2020 were enrolled in the study. The included variables were clinical and demographic characteristics of COVID-19 patients. The case fatality rate (CFR), incidence rates, and R were estimated based on the daily reported data. For estimating R, generation time was assumed on multi scenarios. R was estimated by R0-package. Moreover, Chi square test was applied. All the analyses were performed in STATA, Excel, ArcMap and R. A p-value less than 0.05 was considered as statistically significant.

**Results:**

**Conclusion:**

As R is slightly high, the risk of epidemic has reduced gradually. However, observing social distance and related guidelines are still recommended.

## INTRODUCTION

The first case of Coronavirus Disease (COVID-19) was reported in Iran on February 19, 2020, in Qom province, 150 km south of the capital city of Tehran. Several days later, Markazi province, 140 km west of the Qom, where the current study was performed, was introduced as the second place where the case of COVID-19 was reported. ^1 , 2^

As COVID-19 has imposed a heavy human life and financial burden on countries and threatened their health system, identifying the characteristics of the infected people, its fatality rate, and reproduction number are very important. On one hand, performing these types of studies provides the background to present better healthcare services and better public health response. On the other hand, designing cost-effective vaccines and treatments needs previously identified characteristics of the infected people. ^3^ These issues have necessitated identifying characteristics and basic reproduction numbers of COVID-19 cases. Lastly, our knowledge is little regarding COVID-19 in the city and country. The results of this study have many implications for the global level, too. 

There previously have been guidelines on protecting healthcare workers (HCWs) when exposed to patients, especially the infected ones. ^4^ However, HCWs may have decreased the use of these guidelines due to low supervision and decrease in contagious diseases. This issue has caused a great deal of morbidity and mortality among HCWs. As asymptomatic cases have the main role in transferring COVID-19, the importance of observing these protective guidelines has increased. In this regard, the current study aimed to assess the infection rate among HCWs and compare it with other countries. Healthcare systems which have already been challenged with low human resources will be more challenged with high infection and death among HCWs. ^5^

One of the ways to identify the contagiousness and transmissibility rate of an epidemics is calculating the reproduction number. This index makes it possible to predict an infectious disease transmission, the effectiveness othe f evaluation of the performed measures, and lastly the results of policies taken to control it. ^6^

As reproduction number of COVID-19 is variable in different populations and periods, Iranian researchers have modelled the transmissibility of the COVID-19 virus in different regions. In this regard, some studies have estimated it in Qom city ^7^ and western Iran ^8^ and accordingly have proposed the required preventive and control measures. However, this province is placed among the first provinces that have reported the cases of COVID-19; also, due to its geographical position that connects many provinces to each other, it can be introduced as one of the main COVID-19 epicenters in Iran. Therefore, the current study was performed to assess the characteristics and basic reproduction numbers of COVID-19 cases in the center of Iran, Markazi province. 

## MATERIALS AND METHODS

This is a cross-sectional study conducted in Markazi province of Iran. Markazi province with a population of about 1,430,000 is located in central Iran. The data on 2430 confirmed cases of COVID-19 from Feb 20 to Aug 26, 2020 were used in this study. There are 3 medical universities in Markazi province, among which Arak University of Medical Sciences (ARAKMU) is the largest one that covers 9 cities with a population of about 977,013. ^9^ This study was performed using the secondary data in the regions covered by ARAKMU (Arak, Delijan, Shazand, Khondab, Ashtian, Farahan, Komijan, Mahallat and Tafresh cities) and all COVID-19 confirmed cases were enrolled. There are 2 specialized hospitals for COVID-19 cases in Arak city (Amir-al-momenin and Aiatollah Khansari hospitals), the capital of Markazi province with a population of about 526,182, and 1 specialized ward in each of 8 public hospitals located in the other 8 cities. COVID-19 epidemic started from 20 Feb 2020, in the regions covered by ARAKMU. ^10^

Inclusion criteria were having the residence in regions covered by ARAKMU and giving the Polymerase chain reaction (PCR) test to confirm COVID-19. All the included samples that had done PCR test were (10627) among those who had referred to private clinics, specialized COVID-19 hospitals, the cases diagnosed by primary health care (PHC) centers, those identified by tracking the contacts and lastly the cases identified through public screening were routinely referred to the stated hospitals to perform COVID-19 test. Among them, 2430 and 8197 cases were included and excluded, respectively. The results of all tested cases were reported to the ARAKMU center for disease prevention and control (CDC) on a daily basis. The data were obtained from this center. The data reliability was performed by comparing CDC data with the hospitalization and death data of the diagnostic laboratory that indicated 95% consistency. The variable of “sample preparation date” was the basis of calculation. Moreover, the variable of “resident place” was used for the regions covered by the university. The reported places of residence other than the regions covered by ARAKMU were excluded from the final analysis. The cases of COVID-19 were confirmed by reverse transcription polymerase chain reaction (RT-PCR) assays based on the protocol established by the WHO. ^11^

The study variables were sex (female/male), age (lower than 5, 5-15, 16-45, 46-60 and higher than 60 years old), healthcare worker (yes/no), self-reported history of heart disease (Has your doctor ever told you that you have heart disease? yes/no), self-reported diabetes type 2 (Has your doctor ever told you that you have diabetes? yes/no), city of residence (Arak/Delijan/Mahalat/Ashtian/Tafresh/Khondab/Shazand/Farahan/Komijan), area of residence (rural/urban), admission to an intensive care unit (ICU) (yes/no), death (yes/no), and on admission, having fever (yes/no), cough (yes/no), dyspnea (yes/no), myalgia (yes/no), headache (yes/no), diarrhea (yes/no), sore throat (yes/no), runny nose (yes/no), abdominal pain (yes/no), vomiting (yes/no), chest pain (yes/no), symptoms, and admission status (inpatient/outpatient).

Frequencies and percentage of categorical variables and for continuous variables mean and standard deviations (SD) were reported. Chi-square analysis was applied to determine statistically significant differences between categorical variables and admission status.

The estimated case fatality rate (CFR) was obtained by the number of deaths divided by the number of confirmed infections. Furthermore, incidence rates were calculated by dividing the number of diagnosed infections by the number of populations for each studied area.

The R was estimated based on the reported data during the study period. The generation time (GT) is the time between the infection time of an infected person and the infection time of his or her infector. Probability density functions for generation intervals have been an important input for epidemic models and epidemic data analysis. ^12^ For estimating R, GT was assumed on multi-scenarios. Similar to Hwang et al. ^13^ in two scenarios, GT was considered with the assumption that the data have gamma distribution with mean±SD of the first 6±3 and second 4±2 days . R and related 95% confidence intervals (CI=95%) were estimated by exponential growth and or maximum likelihood method. The exponential growth rate in the onset of an epidemic can be linked to the initial reproduction ratio. The exponential growth rate, denoted by “e”, is defined by the per capita change in the number of new cases per unit of time. As incidence data are integer valued, Poisson regression is indicated to estimate this parameter, rather than linear regression of the logged incidence. The reproduction number is computed as R0=1/c(-e) where C is the moment generating function of the generation time distribution. It is necessary to choose a period in the epidemic curve over which growth is exponential. It proposes the use of the deviance-based R-squared statistic to guide this choice. ^14^

**Figure 1:**
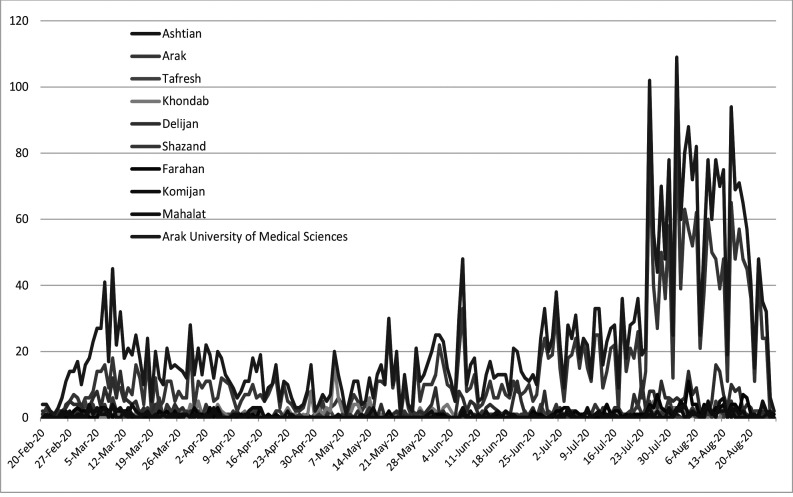
Epidemic curve of COVID-19 in Markazi province separately during 20 Feb to 26 Aug 2020.

Since it is better to calculate R based on the local level, it was calculated in each of the cities covered. The epidemic curves were drawn by the city of residence (Figure 1). Furthermore, R curves were drawn for AUMS and for the center of Markazi province, Arak. All the analyses were performed in STATA 12.0, Excel, version 2003, ArcMap 10.0 and R software, version 3.6.3. A P value less than 0.05 was considered as statistically significant. 

This study was approved by the ethics committee of ARAKMU (IR.ARAKMU.REC.1399.102).

## RESULTS

The results indicated that most of the cases were women with the mean age of 51.78±20.58; most of the cases were in the 16-45 years old age-group. Furthermore, these variables were considered by inpatient and outpatient status of COVID-19 patients in Table 1.

**Table 1:** Descriptive characteristics of Coronavirus Disease (COVID-19) Patients in Arak University of Medical Sciences during Feb 20 to Aug 26, 2020

Variables	N (%)	Admission status		P value*
		Outpatient N (%)	Inpatient N (%)	
Sex	Female	1281(52.70)	412(32.20)	869(67.80)	0.8
	Male	1149(47.30)	365(31.80)	784(68.20)	
Age	lower than 5	18(0.70)	7(39)	11(61.00)	0.001
	5-15	67(2.80)	47(70)	20(30.00)	
	16-45	914(37.60)	467(51)	447(49.00)	
	46-60	559(23.00)	158(28.30)	401(71.70)	
	upper than 60	872(35.90)	98(11.20)	774(88.80)	
History of heart disease	Yes	499(20.50)	73(14.60)	426(85.40)	0.001
	No	1931(79.50)	704(36.50)	1227(63.50)	
History of diabetes type 2	Yes	337(13.90)	46(13.60)	291(86.40)	0.001
	No	2093(86.10)	731(34.90)	1362(65.10)	
City of residence	Arak	1487(61.20)	448(30.10)	1039(69.90)	0.001
	Delijan	297(12.20)	120(40.40)	177(59.60)	
	Mahalat82(3.40)	8(9.80)	74(90.20)		
	Ashtian	28(1.20)	9(32.10)	19(67.90)	
	Tafresh	77(3.20)	53(68.80)	24(31.20)	
	Khondab	148(6.10)	100(67.60)	48(32.40)	
	Shazand	150(6.20)	3(2.00)	147(98.00)	
	Farahan	96(4.00)	20(20.80)	76(79.20)	
	Komijan	65(2.70)	16(24.60)	49(75.40)	
Area of residence	Rural	461(19.00)	138(30.00)	323(70.00)	0.001
	Urban	1969(81.00)	689(35.00)	1280(65.00)	
Fever	Yes	1233(50.70)	321(26.00)	912(74.00)	0.001
	No	1197(49.30)	456(38.10)	741(61.90)	
Cough	Yes	1347(55.40)	340(25.50)	1007(74.80)	0.001
	No	1083(44.60)	437(40.40)	646(59.60)	
Dyspnea	Yes	960(39.50)	138(14.40)	822(85.60)	0.001
	No	1470(60.50)	639(43.50)	831(56.50)	
Myalgia	Yes	653(26.90)	219(33.50)	434(66.50)	0.31
	No	1777(73.10)	558(31.40)	1219(68.60)	
Sore throat	Yes	266(10.90)	93(35.00)	173(65.00)	0.26
	No	2164(89.10)	684(31.60)	1480(68.40)	
Runny nose	Yes	53(2.20)	29(54.70)	24(45.30)	0.001
	No	2377(97.80)	748(31.50)	1629(68.50)	
Diarrhea	Yes	156(6.40)	42(26.90)	114(73.10)	0.16
	No	2274(93.60)	735(32.30)	1539(67.70)	
Vomiting	Yes	257(10.60)	52(20.20)	205(79.80)	0.001
	No	2173(89.40)	725(33.40)	1448(66.60)	
Headache	Yes	448(18.40)	188(42.00)	260(58.00)	0.001
	No	1982(81.60)	589(29.70)	1393(70.30)	
Chest pain	Yes	197(8.10)	69(35.00)	128(65.00)	0.33
	No	2233(91.90)	708(31.70)	1525(68.30)	
Abdominal pain	Yes	134(5.50)	42(31.30)	92(68.70)	0.87
	No	2296(94.40)	735(32.0)	1561(68.0.)	
Death	Yes	205(8.40)	7(3.40)	198(96.60)	0.001
	No	2225(91.60)	770(34.60)	1455(65.40)	
Admission to an intensive care unit (ICU)	Yes	79 (3.30)	1(1.30)	78(98.70)	0.001
	No	2351(96.70)	776(33.00)	1575(67.00)	
Health care worker	Yes	206 (8.50)	143(69.40)	63(30.60)	0.001
	No	2224(91.50)	634(28.50)	1590(71.50)	

* Chi-square test

Regarding background diseases and situations, the frequency distributions of the history of cardiovascular diseases and diabetes are shown in Table 1. The most prevalent symptoms reported by COVID-19 patients were cough, fever and dyspnea. Arak and Delijan cities had the greatest number of confirmed cases ( Table 1). There were statistically significant differences by admission status for age, History of heart disease, History of diabetes type 2, City of residence, Area of residence, fever, cough, dyspnea, runny nose, vomiting, headache, death, Admission to an intensive care unit (ICU) and HCW (P=0.001) ( Table 1).

**Figure 2:**
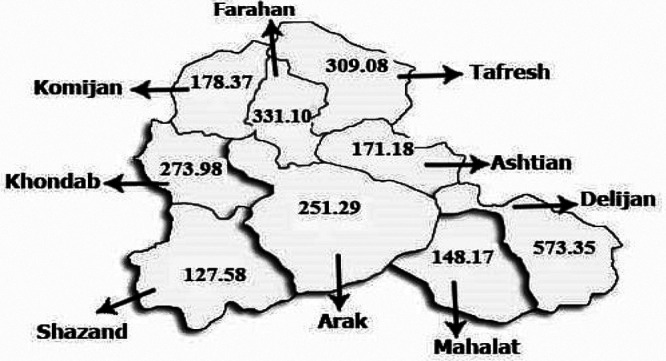
Map of the distribution of COVID-19 cases in the study area

The most incidence rates of COVID-19 per 100,000 persons were located in Delijan (575.35, CI 95%: 510.10, 640.59), Farahan (331.10, CI 95%: 264.98, 397.23), Tafresh (309.08, CI 95%: 240.15, 378.00), Khondab (273.98, CI 95%: 229.90, 318.06), Arak (251.29, CI 95%: 238.54, 264.05), Komijan (178.37, CI 95%: 135.05, 221.70), Ashtian (171.18, CI 95%: 107.83, 234.53), Mahallat (148.17, CI 95%: 116.12, 180.22), and Shazand (127.58, CI 95%: 107.18, 147.99), respectively. CFR in all of the population was relatively high ( Table 1). A map of the distribution of COVID-19 cases in this province is displayed in Figure 2.

**Figure 3:**
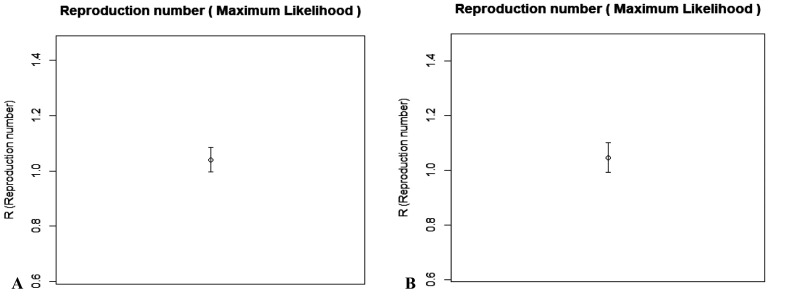
Reproduction number curve for A) districts of Arak University of Medical Sciences R=1.04 (1.00, 1.08), Mean and SD of Gamma Distribution for Generation time 6 and 3, respectively. and for B) The center of Markazi province; Arak, R=1.05 (0.99, 1.10), Mean and SD of gamma distribution for generation time 6 and 3, respectively.

**Figure 4:**
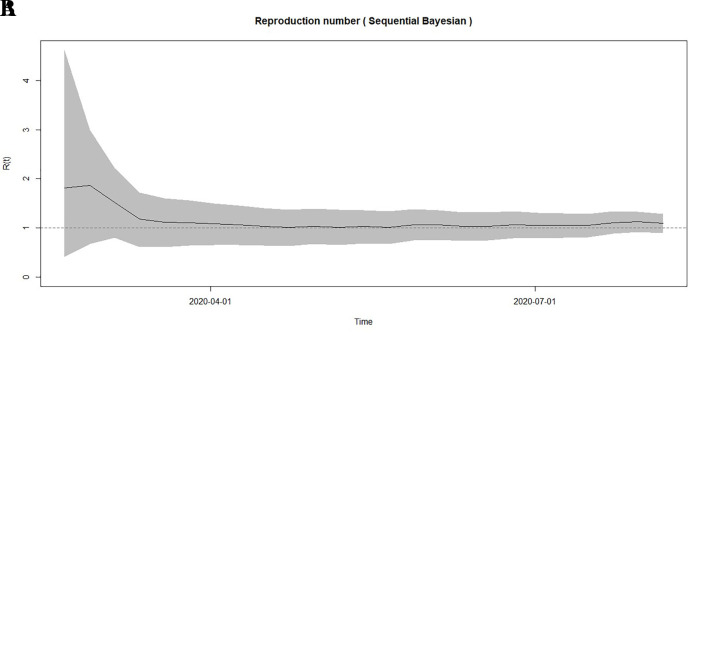
The changes of reproduction number by week from 20 Feb, to 26 Aug, 2020 in A) Districts of Arak University of Medical Sciences and B) The center of Markazi province; Arak.

The R on the basis of the GT has been calculated on several scenarios in two times: first, in March 19, and second in August 26, 2020. Accordingly, on the basis of 2 GT scenarios calculated, in the first time, R was lowest in Komijan city and highest in Arak and Khondab cities. ( Table 2). The highest to the lowest R values were observed in Arak, Khondab, Ashtian, Farahan, Mahallat, Delijan, Shazand, Tafresh and Komijan cities, respectively. However, it is notable to state that the difference of R from 1 was statistically significant only in Arak and Delijan cities. The R lower than 1 means that the epidemic place in the control step. R equal to 1.5 (the amount calculated for Arak city) indicates that, for example, 10 infected people can infect 15 susceptible persons. By administering an interventional program with 100% effectiveness with this R, it is expected that if 33% of the population is covered by that intervention, the number of R will be lower than 1. Also, by administering an interventional program with this R and 50% and/or 33% effectiveness, it is expected that if 66% and/or 100% of the population are covered by that intervention, the amount of R will be lower than 1. Moreover, R equal to 1.5 means that the probability of effective contact is 1.5%. This amount leads to a gradual epidemic that will infect 60% of the population ( Table 2, Figures 1, 3 and 4).

**Table 2:** Generation time and reproduction number (R) of COVID-19 in Arak University of Medical Sciences during Feb 20 to Aug 26, 2020

City	Generation Time	Mean±SDR* (CI 95%) (Feb 20 to Mar 19, 2020)	R** (CI 95%) (Feb 20 to Aug 26, 2020)
Arak	6±3	1.48 (1.32, 1.67)	1.05 (0.99, 1.10)
Arak	4±2	1.32 (1.21, 1.44)	1.02 (0.97, 1.07)
Delijan	6±3	1.16(1.01, 1.34)	1.03 (1.02, 1.04)
Delijan	4± 2	1.11 (1.00, 1.23)	1.02 (1.01, 1.03)
Mahalat	6±3	1.21 (0.94, 1.55)	1.01 (0.98, 1.03)
Mahalat	4±2	1.14 (0.96, 1.36)	1.00 (0.99, 1.02)
Ashtian	6±3	1.38 (0.89, 2.15)	1.04 (1.01, 1.07)
Ashtian	4±2	1.26 (0.92, 1.73)	1.03 (1.00, 1.05)
Tafresh	6±3	1.09 (0.76, 1.53)	1.05 (1.03, 1.07)
Tafresh	4±2	1.06 (0.83, 1.35)	1.03 (1.02, 1.05)
Khondab	6±3	1.48 (0.83, 2.69)	1.00 (0.99, 1.02)
Khondab	4±2	1.31 (0.88, 2.03)	1.00 (0.99, 1.01)
Shazand	6±3	1.14 (0.82, 1.56)	0.99 (0.97, 1.01)
Shazand	4±2	1.09 (0.87, 1.37)	0.99 (0.98, 1.00)
Farahan	6±3	1.22 (0.83, 1.76)	1.08 (1.06, 1.10)
Farahan	4±2	1.15 (0.88, 1.49)	1.05 (1.04, 1.07)
Komijan	6±3	0.69 (0.69, 1.42)	1.02 (0.99, 1.04)
Komijan	4±2	NA	1.01 (1.00, 1.03)
Arak University of Medical Sciences	6±3	1.28(1.19, 1.37)	1.04 (1.00, 1.08)
Arak University of Medical Sciences	4±2	1.19(1.13, 1.25)	1.04 (1.04, 1.05)

* R based on Exponential Growth Method;

** R based on Maximum likelihood Method

In the second time, R was lowest in Shazand city and highest in Farahan city ( Table 2). The highest to lowest R values were observed in Farahan, Tafresh, Arak, Ashtian, Delijan, Komijan, Mahallat, Khondab and Shazand cities, respectively. However, it is noteworthy to mention that the difference of R from 1 was statistically significant only in Farahan, Tafresh, Ashtian and Delijan cities ( Table 2).

## DISCUSSION

Markazi province was the second province where the first case of COVID-19 was found. However, the number of cases was decreased following the performance of telephone screening of COVID-19 suspected cases using PHC system on the basis of the symptoms of dry cough, fever, and dyspnea. 

The study indicated that morbidity of COVID-19 varies in the cities located in Markazi province. Among the reasons are difference in demographic variables, distance from the epicenter city, restrictive policies, and the status of COVID-19 epidemiology in those cities. 

Regarding demographic variables, this study indicated that the greatest number of inpatient cases were among the elderly. A study in Iran indicated that higher percentage of deaths occurred among the elderly. ^15^ Also, another study in Iran indicated that most of the cases and the severe ones occurred among the patients over 50 years old and those with background diseases. ^16^ The evidence in Italy indicates that as its population is older than other countries, it has higher morbidity and mortality. Due to having comorbidities including hypertension, diabetes and chronic respiratory diseases, the elderly have a higher morbidity and mortality. However, the selected strategy to deal with the patients is highly effective on the number of deaths. ^17^ This study is in the same line with other studies in Iran, indicating that a greater number of positive cases occur among the women. ^15 , 18^ It has been proven that women due to their chromosomal status ^19^ and also special women hormone, estrogen, ^20^ have higher resistance and adaptability to more severe cases than men. Thus, although this study showed almost equal morbidity between women and men, the number of severe cases and deaths is higher among men. 

The results indicated that a higher percentage of the cases are among upper middle-ages and the elderly. The cases with severe diseases and hospitalized in the ICU units were those with higher ages and background diseases. A Chinese study confirmed that most patients hospitalized in the ICU units had higher ages and comorbidities than patients not admitted to the ICUs. ^21^ The results indicated that one fifth of the cases had a history of cardiovascular diseases and then diabetes type 2. A study in Wuhan indicated that half of the COVID-19 cases had one or more comorbidities. Accordingly, blood pressure, malignancy, diabetes and cardiovascular diseases are among the most important comorbidities, respectively. ^21^

This study indicated that the most prevalent COVID-19 symptoms reported are cough, fever and dyspnea, respectively. The present guidelines on COVID-19 published by the healthcare system authorities indicate that the most prevalent symptom is dyspnea. However, a systematic review indicated that a low percentage of the patients had this symptom. ^22^ The current study indicate that dyspnea is the third most prevalent COVID-19 symptom. 

The results indicated that a high percentage of the cases were among HCWs. Morbidity and mortality among HCWs have always been of interest. A high percentage of the confirmed COVID-19 cases in China have been among HCWs, and a considerable number of them have died. ^23^ Also, a high percentage of the COVID-19 cases in Italy was among HCWs and about half of them have died. ^24^ One fifth of the cases in Lombardy, Italy, were also among HCWs. ^25^ This rate was higher in Spain. ^26^ Morbidity and mortality rate due to the previous coronavirus outbreaks such as severe acute respiratory syndrome (SARS) and Middle East respiratory syndrome (MERS) have been high. ^27 , 28^ Despite cautions and guidelines on the high transferability of nosocomial infections, high morbidity and mortality have happened among HCWs due to not taking these precautions seriously. In this regard, countries should distribute healthcare protective items such as face masks in the community with the priority of HCWs, so that there is healthy HCWs to provide services against COVID-19. HCWs that provide services to COVID-19 patients are faced with high stress, burnout and low working capacity. Performing the Productivity Improvement Act can provide adequate support for them and promote workforce productivity, quality of care, increase in the morale of HCWs, decrease in the problems related to work plan development, and equity in the payments. ^29^

CFR index has many heterogeneities in different regions of the world. It has been reported lower in the African region and higher in the East Mediterranean region compared to the results of the current study. There have been many causes for this difference. First of all, the Corona test is not done for everyone and secondly many of the infected people indicate mild symptoms of the disease that are not reported as positive cases. Another issue is the population size and background diseases. Accordingly, it has been proven that age, gender, and background diseases have a direct relationship with CFR. ^30 , 31^ A study on COVID-19 in Wuhan, China, indicated that CFR was higher among the elderly. ^32^

The results indicated that the number of cases in the studied cities decreased in a similar time frame that is indicative of similar policies on restrictions, social distancing, and media campaigns. The case of China also indicated that the number of cases after a period of disease outbreak decreased following restrictive measures. ^33^

In the months of the onset of epidemic, it is necessary for the cities to impose many restrictions on the entry of passengers from the cities that are most affected. Japan, as the third country that has been affected by this disease, has been successful in controlling it by imposing severe restrictions on the entry of passengers from China to Japan, establishing active care, following the contacts, and using different guidelines. However, the implementation of strict travel restrictions without public health policies and behavior change has a limited effect on the disease transmission. ^34^

This study, along with the strengthens, had some limitations. Although CFR calculation in this study has many implications, it has been considered very simple. Valid calculation of CFR needs the identification of all positive cases. However, this is almost impossible due to the large number of asymptomatic patients. ^10^ In other words, as the most reported confirmed cases in this study were among the patients with severe forms of the disease, it should be noted in all of the interpretations that the calculated values are minimal amounts and the actual values may be much higher. Many of the infected persons are asymptomatic and can transmit the disease to others. ^35^ Thus, it is recommended that CFR should be calculated again after the final determination of the cases. Moreover, since a number of asymptomatic patients were not included in this study, potentially there was under-reporting bias for the findings of this study.

Reproduction number or R is an important index to estimate the transmissibility of the COVID-19 outbreak. The amount of R obtained in the onset of the study period was meaningfully lower than other countries’ estimations. ^32 , 36 , 37^ Low R value indicates that governmental policies on observing social distancing, travel prevention and closure of universities, schools, industries, and jobs have been successful. Another study in Iran, confirming the findings of this study, states that R has a decreasing trend.38 The most important limitation of this study was lack of considering other influential variables on COVID-19 morbidity; so further studies are recommended to be conducted in this regard. 

## CONCLUSION

Although the COVID-19 outbreak is decreasing in Markazi province, it is necessary to update the characteristics of the infected patients in order to determine the changes in the virus strains. COVID-19 high morbidity and mortality among HCWs are warning signs for healthcare authorities to pay close attention to the protective guidelines in health system facilities. Absolutely, sharing this information among the authorities and policymakers provides the basis for prevention and control of the disease. Performing the same policies on prevention and control of COVID-19 in the levels of cities and provinces provides reciprocal benefit for them. Considering the successful programs on screening and identifying asymptomatic people, it is recommended that their implementation should continue.
